# Bioactive Virus-Mimicking Nanovesicles From Dendrimersomes: A Novel Approach to Understanding SARS-CoV-2 Host-Interactions to Better Design Therapeutics

**DOI:** 10.3389/fmolb.2020.00188

**Published:** 2020-08-21

**Authors:** Bilal Javed

**Affiliations:** ^1^Roy and Diana Vagelos Laboratories, Department of Chemistry, University of Pennsylvania, Philadelphia, PA, United States; ^2^Faculty of Sciences, PMAS Arid Agriculture University, Rawalpindi, Pakistan

**Keywords:** COVID-19, dendrimers, immune response, pseudoviruses, surface modifications, therapeutic carriers, vaccine

Surface modified bioactive virus-mimicking organic nanovesicles represent themselves as complex biomimetic structures that have abilities to dissect the functions of individual entities in a complex biochemical system. The customized surface of nanovesicles from (glyco)dendrimersomes has structural modifications that contribute to manifest the SARS-CoV-2 and host pathogenic molecular interactions that help the virus to evade the human immune system. Nanovesicles also have a potential role in dynamic biomedical applications such as designing galectin targeting drugs, evaluation of antibodies to design antiviral therapeutics or vaccines and targeted delivery of drugs.

## Introduction

Molecular and biochemical dynamics of the plasma cell membrane and other structural coatings provide a great insight to design customized structural components of biomimetic nanovesicles by using techniques of organic supramolecular chemistry and have abilities to self-assemble in the form of spherical vesicles upon injection to an aqueous environment and are called dendrimersomes. Dendrimersomes represent themselves as multi-dynamic, bioactive, synthetic, and hybrid cell-like or virus mimicking surface modified nanovesicles that help scientists to understand the molecular mechanisms of host-pathogen interactions to better design therapeutics (Sherman et al., 2017). Dendrimersomes have advantages on other routine biochemical and pseudovirus (retroviruses pseudo-typed with SARS-CoV-2 spike proteins) entry assays because of their stability *in vitro*, biocompatibility and customized design that provides a better understanding of a particular function with safe handling. Designing spike-like oligomannoses of SARS-CoV-2 on the surface of (glyco)dendrimersomes play a significant role to translate host-pathogenic molecular mechanisms (Brockmeier, 2020). This opinion article presents an idea to use sequence-defined surface modified (glyco)dendrimersomes to better understand individual pathological features in a multiplex SARS-CoV-2 structure. It also explains the significant role of (glyco)dendrimersomes or nanovesicles to translate glycan and galectin interactions that can help to design galectin targeting drugs to control viral infections.

## Sequence-Defined Synthetic Virus-Mimicking Nanovesicles From (glyco)dendrimersomes

**Figure 1:**
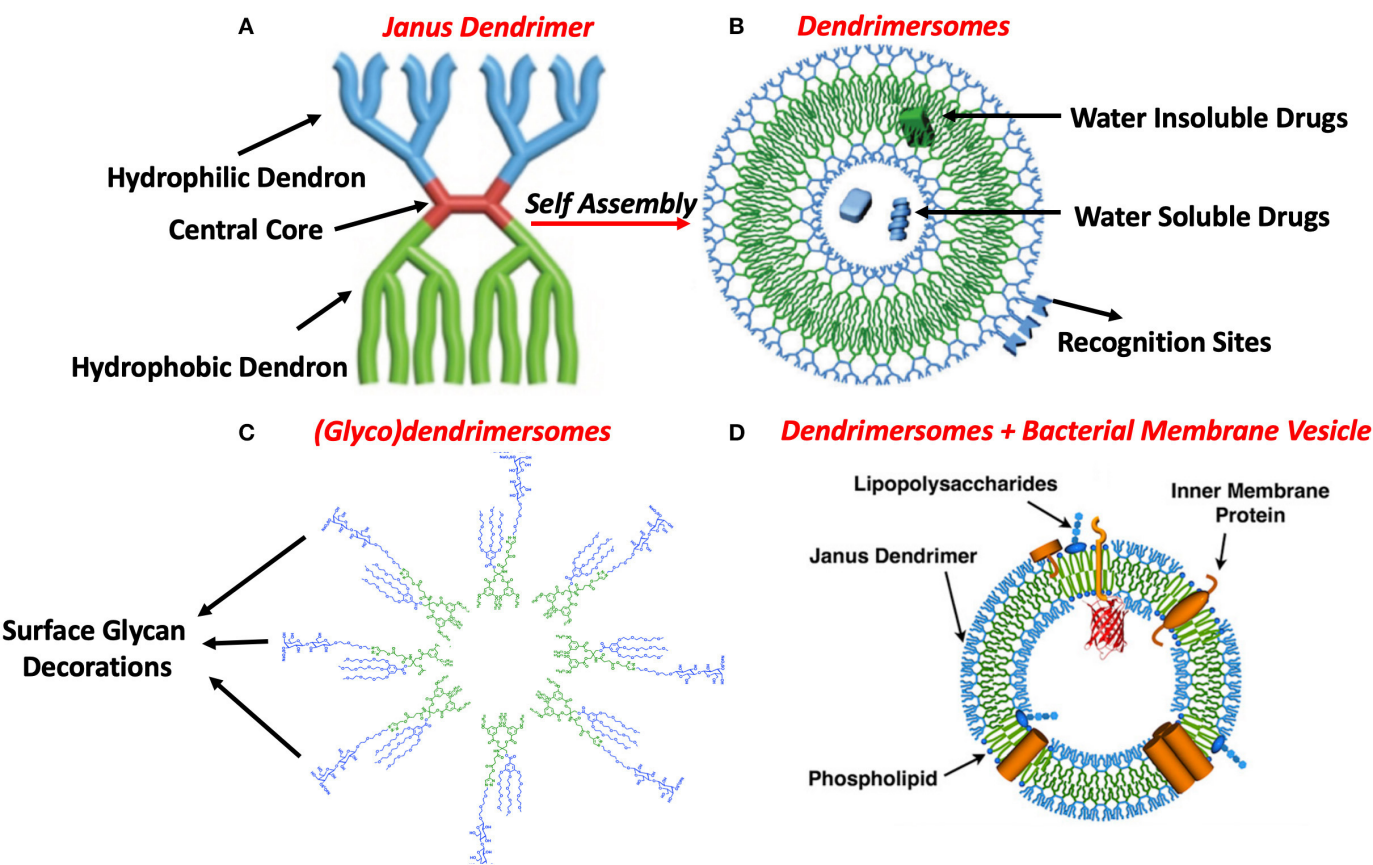
**(A)** Janus dendrimer having a central core (Red), hydrophilic dendron (Blue) and a hydrophobic dendron (Green), act as a structural unit of dendrimersomes. **(B)** Self-assembled bioactive dendrimersomes as a biomimetic nanovesicle to encapsulate hydrophilic and hydrophobic therapeutics, surface modifications include recognition sites that help dendrimersomes to bind to their targets. **(C)** Structure of a glycodendrimersomes having bioactive customized glycans on their surface helps to decode glycan-galectin interactions. **(D)** A hybrid nanovesicle having dendrimers and structural components of the bacterial membrane provide advantages to carriage therapeutics and act as vaccines to neutralize future pathogenic invasions. The figure is adapted from Xiao et al. (2016a, 2018) and Sherman et al. (2017) and modified as required. Color Scheme: Green; *Hydrophobic*, Blue; *Hydrophilic*.

Dendrimers are supramolecular amphiphilic assemblies, have both hydrophilic and hydrophobic units which are called dendrons and arrange themselves on a central core and make a symmetrical structure called dendrimersomes (Figure 1A) (Xiao et al., 2016b). These dendrimersomes represent themselves as bioinspired membrane mimics that have a customizable sequence of proteins and sugars called glycoproteins on their outer surface and encloses an empty lumen or pocket (Figure 1B) (Sherman et al., 2017).

Sequence defined surface modification of (glyco)dendrimersomes with glycans and glycoproteins (oligomannoses) found on the surface of viruses including SARS-CoV-2 and other living cells or bacterial pathogens provide an opportunity to understand molecular interaction patterns between host and pathogen. This customized system helps to better understand the effects of one single molecular function at a time rather than to attain a synergistic response which becomes difficult to explain (Brockmeier, 2020).

It is challenging to handle wild-type viruses that require special biosafety level 3 or 4 containment laboratories. However, the use of pseudoviruses in place of wild-type viruses provide advantages to remove virulent genes from viral nucleocapsid and handle them in biosafety level 2 laboratories to better develop antivirals agents or vaccines. Importantly, the envelopes of pseudoviral particles may have similar conformational structures or envelop to those of the wild-type viruses, making it feasible to conduct a mechanistic investigation on viral entry and to evaluate potential neutralizing antibodies. However, certain challenging issues still exist such as including the production of a sufficient pseudovirus yield and the inability to produce an appropriate pseudotype of certain viruses and understanding synergistic mechanistic response (Li et al., 2018). A novel alternative approach involves the decoration of spike-like S oligomannoses on the surface of (glyco)dendrimersomes to better understand host-pathogenic molecular mechanistic interaction. Designing an epitope uniformly on the surface of vesicle manifests singular molecular function and provide a better understanding. The customized design of virus-mimicking nanovesicles has advantages to understand one biomolecular mechanism at a time in a complex viral system (Xiao et al., 2020).

The presence of the surface exposed oligomannose spike S glycoproteins on the surface of the SARS-CoV-2 help them to interact with angiotensin-converting enzyme 2 (ACE2) receptor on the surface of the host cells and mediates entry inside cells (Wang et al., 2020; Xiao et al., 2020). The special features of the spike S proteins of SARS-CoV-2 make them different from SARS-CoV and provide abilities to evade the host immune system. S glycoproteins are the main targets to understand the host-pathogen interactions and provide functional epitopes for neutralizing antibodies to better design therapeutics or vaccines by using (glyco)dendrimersomes (Shang et al., 2020).

## Bioactive Cell-Like Programmable glycodendrimersomes to Design Galectin-Targeting Antiviral Therapeutics

Galectins are protein in nature and are small endogenous sugar receptors usually targeting ß-galactoside epitopes and control multiple physiological responses and disease-associated signals. Because of the significant contribution of galectins into various pathophysiological functions, galectins have the focus of many viral therapies that are difficult to contain by using conventional antiviral agents (Zhang et al., 2015b; Dings et al., 2018). However, it is very important to understand the interaction of the galectins with the glycoconjugates (glycans) that exist on the seminal fluid mosaic plasma membrane (Rodriguez-Emmenegger et al., 2019). It is not possible to decode glycan and galectin interactions by using live cells due to complex cell structure, glycome variations among the similar type of cells and difficulties to handle and maintain cells outside the body (Zhang et al., 2015a).

Click chemistry provides an advantage to decorate glycoproteins on the surface of (glyco)dendrimersomes that have customized sequence of sugars or proteins and allow scientists to understand how host and pathogens interact? and How intercellular signaling changes during the process of disease? (Brockmeier, 2020). It was better explained previously by using synthetic cell-like glycodendrimersomes that the customized short-chain sugar (glycans) modifications on the surface of glycodendrimersomes act as communication channels and also contribute to recognizing between cells. Understanding of communication or signaling between host and pathogen plays a vital role to design vaccines and other therapeutics (Rodriguez-Emmenegger et al., 2019).

Synthetic cell-like glycodendrimersomes also provide advantages to understand the underlying interaction mechanisms between glycans decorated on the surface of glycodendrimers with human galectins which are not possible to manifest by using natural cells (Figure 1C). Glycan and galectin interactions result in the transfer of signals between cells and also involve in cell recognition that alters during disease conditions (Zhang et al., 2015b). Galectin-inhibiting antiviral drugs are still in infancy due to complex galectin structure, mutations and underlying signal transmissions patterns. The use of sequence defined glycodendrimersomes provide a novel platform to understand signaling during disease, transmission patterns and designing galectin-targeting antiviral medicines (Kopitz et al., 2017).

## Hybrid Cell-Like or Virus-Mimicking Nanovesicles From (glyco)dendrimersomes to Overcome the Immune Response

Hybrid nanovesicles include both customized synthetic amphiphilic dendrimers and components of the plasma cell membrane from bacterial or human cells or it can also incorporate structural units of viral capsid (Xiao et al., 2016a; Yadavalli et al., 2019). Hybrid nanovesicles provide advantages against immune response and have better immune adjustments because of the presence of the components of the body cells (Figure 1D). Cell-like hybrids of customized (glyco)dendrimersomes and pathogenic membranes provides a combination of a chemical and biological membrane in one entity. Virus-recognizing modifications on the surface of hybrid virus-mimicking dendrimersomes act as an epitope and help to neutralize or defunctionalize virus binding to human ACE2 receptors. While the purpose of the incorporation of components of the cell membrane is to help them to better adjust or recognized by the immune system of the human body. Such tunable cell-like hybrids with custom-made combinations of surface epitopes and active receptors will likely find utility in dissecting the functionality of individual entities in complex networks and ultimately enable novel biomedical applications (Xiao et al., 2016a).

## Sequence-Defined Biomimetic (glyco)dendrimersomes to Attach to Host Recognition Sites and Designing Vaccines

Programmable/customized glycoprotein decorations as the terminal groups on the surface of bioactive virus-mimicking (glyco)dendrimersomes also have abilities to act as an epitope of an antigen and to be recognized by the antibodies secreted by immune cells to generate an immune response and neutralize the future pathogenic attacks. These (glyco)dendrimersomes also act as a functional platform to screen antibodies to design and select vaccines. Empty lumen or pocket of the (glyco)dendrimersomes provide an advantage to use them as a carrier of antiviral therapeutics that can target specific virus particles because of their surface modifications and release drugs upon exposure to a particular physicochemical stimulus such as ultra-violet light. Surface targeting and controlled release of drugs have advantages to better manage patients' health and have profound treatment success rates (Li et al., 2020).

Some previous studies expressed (glyco)dendrimersomes as nearly safe against human umbilical vein endothelial cells (HUVEC) and they were observed viable upon exposure to nanovesicles that require further toxicological investigations to use them as drug carriers (Sherman et al., 2017).

## Future Perspectives/Summery

Designing therapeutics requires a clear understanding of host-pathogenic molecular interactions that involve the nature, mechanism of action and transmission patterns of SARS-CoV-2 which are difficult to understand by using wild-type of pathogens. Sequence-defined virus-mimicking nanovesicles provide opportunities to study the functions of individual entities in complex biochemical systems. As scientists are working worldwide to design therapeutics to contain the further transmission of the viral outbreak; bioactive virus-mimicking synthetic and hybrid (glyco)dendrimersomes provide a multi-dynamic platform to understand host-pathogen interactions that help SARS-CoV-2 to evade the human immune system. Nanovesicles have the potential to be used as a vaccine having customized oligomannoses as terminal groups on their surface that represent the blueprints of spike S proteins. The empty lumen of organic supramolecular vesicles has abilities to carriage antiviral drugs or therapeutics to their targeted sites or pathogens and release drugs in response to a particular stimulus.

## Author Contributions

BJ designed the study and wrote the manuscript.

## Conflict of Interest

The author declares that the research was conducted in the absence of any commercial or financial relationships that could be construed as a potential conflict of interest.
